# Measuring Animal Welfare within a Reintroduction: An Assessment of Different Indices of Stress in Water Voles *Arvicola amphibius*

**DOI:** 10.1371/journal.pone.0041081

**Published:** 2012-07-17

**Authors:** Merryl Gelling, Paul J. Johnson, Tom P. Moorhouse, David W. Macdonald

**Affiliations:** Wildlife Conservation Research Unit, Department of Zoology, University of Oxford, Recanati-Kaplan Centre, Tubney, United Kingdom; Australian Wildlife Conservancy, Australia

## Abstract

Reintroductions are an increasingly common conservation restoration tool; however, little attention has hitherto been given to different methods for monitoring the stress encountered by reintroduced individuals. We compared ten potential measures of stress within four different categories (neuroendocrine, cell function, body condition and immune system function) as proxies for animal welfare in water voles being reintroduced to the Upper Thames region, Oxfordshire, UK. Captive-bred voles were assessed pre-release, and each month post-release for up to five months. Wild-born voles were captured in the field and assessed from two months post-release. Plasma corticosteroid, hydration and body condition of captive-bred voles differed between their pre-release measures and both their first (“short-term”) recapture, and their final recapture (“long-term” release), however only body condition and immunocompetence measured using the Nitroblue Tetrazolium (NBT) test were significantly different post-release between the first and last recaptures. Captive-bred animals had lower fat reserves, higher weight/length ratios and better immunocompetence (NBT) than did wild-born voles. Captive-bred males had higher ectoparasite burdens compared to wild-born males and, as reintroduction site quality decreased, became less hydrated. These observations indicate that some methods can identify changes in the stress response in individuals, highlighting areas of risk in a reintroduction programme. In addition, a single measure may not provide a full picture of the stress experienced; instead, a combination of measures of different physiological systems may give a more complete indication of stress during the reintroduction process. We highlight the need to monitor stress in reintroductions using measures from different physiological systems to inform on possible animal welfare improvements and thus the overall success rate of reintroductions.

## Introduction

Wildlife reintroductions aim to re-establish viable populations of a species within their indigenous home-range following local extinction or extirpation using translocated or captive-bred individuals [1], [2]. They are often only partially successful [3], [4], [5], [6]. One plausible explanation advanced for this overall lack of success is ‘stress’, and thus the impact of stress on reintroduction success is a research priority [6], [7], [8], [9]. Measures of stress can provide an assessment of animal welfare [10], therefore measuring stress in wildlife is of increasing interest to conservationists to establish the physiological effects of anthropogenic factors [4], [5], [6], [7], [8], [9].

‘Stress’ can be defined as ‘the biological response elicited when an individual perceives a threat to its homeostasis’ [11]. The reactive processes which deal with everyday events are adaptive responses [6].Nevertheless, sustained acute [12] or chronic stress may result in the diversion of resources to cope with the stressor [11]. Stress may have positive or negative effects on the immune response. Viswanathen et al. [13] found that exposure to acute transient stressors promoted immune enhancement, while chronic stress and long-term immune over-activity were immunosuppressive, potentially resulting in pathology. Individuals may ultimately incur significant biological costs, including adverse effects on growth and development, reproduction, or pathogen resistance [6], [14], [15]. Once a stressful situation has been overcome using physiological responses, normal physiological functioning should resume. However stress can induce an individual into a pre-pathological state involving the development of morbidity, a clear indication that an individual has moved past the maintenance of a homeostatic stress response into a state of distress [11], which may influence survival. Macdonald [16] cites animal welfare as one of seven potentially ‘awkward questions’ for reintroduction, and advocates incorporating animal welfare science into conservation biology as a likely means of improving reintroduction success. The IUCN [2] reintroduction guidelines state that every translocation must meet mandated animal welfare standards during all stages of the project, with the aim of reducing stress or suffering whenever possible. When animals are captive-bred and released, or wild-born, measures of the stress response are urgently required [17].

Individuals may be stressed at any stage of reintroduction, including while in captivity (whether translocated or captive-bred; [18]), in pre-release housing [7], during transportation [19], [20], the release itself [21], and post-release (e.g. [17], [22], [23]. Many different parameters have been suggested as possible indicators of stress and immunocompetence within the wider literature, and might therefore be used as proxies for animal welfare. Nonetheless, Harrington et al. (unpublished data) reviewed 199 published reintroduction projects finding that only three projects reported stress measures - two using faecal corticosteroids and one using white blood cell ratios –18% took some measure of body condition, usually weight during post-release live-trapping, and only one measured bacterial infection and changes in faecal parasite load.

We explore a range of biological parameters as indicators of animal welfare, using a water vole *Arvicola amphibius* Linneus reintroduction program in the Upper Thames region, Oxfordshire, UK. The water vole has declined in the UK, with numbers estimated to have fallen by up to 95% since the 1960s, due both to habitat loss and introduction of the invasive American mink *Neovison vison* [24]. Where the causes of decline have been remedied, reintroduction is likely to be an important component of future conservation efforts [25], [26].

We aimed to identify and assess different measures of stress to identify patterns to indicate ways in which these measures, either individually, or in combination, might be applied to the monitoring of animals undergoing reintroduction.

### Candidate Measures of Stress

Moberg [10] identified three biological systems that cope with stressors: behaviour, the autonomic nervous system, and the neuroendocrine system. The neuroendocrine system is regularly monitored in wild populations and biological and immunological factors may provide additional information on the welfare status of each individual.

#### Neuroendocrine

The neuroendocrine system regulates glucocorticoid release from the adrenal gland [27] and is activated by the hypothalamic-adrenal-pituitary (HPA) axis [22]. In most vertebrate species corticosteroid levels can be measured in faeces, urine and serum [27]. Corticosteroids are often considered ‘the’ stress hormones (e.g. [28]). However, numerous factors affect the corticosteroid response, including time of day, season, breeding condition and feeding [29]. These affect both serum (acute) and faecal (chronic) corticosteroid levels. Nonetheless, there are benefits to using faecal corticosteroid: it may be collected non-invasively, and does not therefore necessitate trapping or handling [30], [31].

#### Cell function

Cells are maintained by a hydration shell [32]. Hydration status affects cell function [33] and might therefore indicate chronic stress. Moorhouse et al. [9] have shown that prolonged captivity under sub-optimal housing conditions in water voles (when housed in laboratory cages rather than outside enclosures) decreases their hydration levels, regardless of *ad libitum* access to water.

#### Condition indices

Body condition indices of vertebrate species have been measured by fatty body mass resources available (e.g. [34], [35]), an index of weight/length ratio (e.g. [36]) and simply by changes in individual weight (e.g. [37]). Reduced fat stores are indicative of chronic stress by indicating ability to transpose energy into growth and condition, as opposed to maintaining immunocompetence.

#### Immune system function

Immunocompetence challenge techniques include *in vitro* measures of immunocompetence and have proved a successful tool in wild mammal studies (e.g. [9], [19], [38]). The Nitroblue Tetrazolium (NBT) test is an immunocompetence challenge test that measures neutrophil activity, which is associated with acute stress activation [19], [39] and is easily used on small samples of peripheral blood. Additionally a simple score of ectoparasite burden can provide an indication of stress levels and immunocompetence [40].

Chronic immunological challenges may influence morphological pheno-deviance (morphological abnormalities not associated with a particular genotype or trait), which has been linked to poor developmental homeostasis [41]. Under normal conditions, vertebrates are expected to develop bilateral traits (e.g. leg length) symmetrically [23]. Deviation from normal developmental conditions may result in developmental instability, expressed by fluctuating asymmetry [42].

In this study, we compare changes in the stress response of captive-bred and reintroduced adults over time, both in the short-term (one month post-release) and long term (either four or five months post-release) hypothesizing that the biggest difference in the stress response will occur in the early stages of reintroduction as the individuals acclimatize to their new environment. We then compare captive-bred and subsequent wild-born adults, hypothesizing that captive-bred animals responding differently to those wild-born indicates potential disadvantages arising from their captive-bred status, which in turn might impact upon the overall success of the reintroduction.

## Materials and Methods

### Reintroduction Protocol

Water voles were reintroduced into 12 sites along rivers within the Upper Thames region, UK (see [25], [43] for site details and methodology followed) over three years (2005–2007 inclusive). The voles were bred in captivity late during the previous year and housed in sibling groups in outdoor enclosures, before being transported to Oxford. They were then maintained in captivity in Oxford in smaller, same-sex sibling groups, for a maximum of 18 days before release.

At six of the reintroduction sites, blood, feces and urine were collected from voles pre-release, as well as once a month for up to five months post-release. Five months is appropriate for monitoring this species, given that water voles’ breeding seasons typically last seven months during which time a female may have up to five litters [26]. Data were collected on ten measures of stress, which were separated into four main categories; neuroendocrine (plasma and faecal corticosterone); cell function (urine refractive index and urine specific gravity); body condition (fat reserve index, weight/length ratio and weight) and immune system function (NBT, parasite score and fluctuating asymmetry).

### Sampling Protocol

Before release, each individual was anaesthetised with an inhalation mixture of oxygen and isoflourane (2%) delivered at a rate of 2 l min^−1^ (Isocare, Animalcare Ltd, York, UK; [44]. Post release, animals were re-captured - using Sherman XLK 8×9×30 cm (H.B.Sherman Traps Inc., Tallahassee, F.C, U.S.A.) folding aluminium live traps with galvanized steel doors, baited with carrot and apple, and placed at 15 m intervals along the banks of the water course (see [43] for details), anaesthetised once more, and resampled. Traps were set from 16∶00 hrs each day and checked from 08∶00 hrs each morning; empty traps were shut during the day to preclude daytime captures. Biometric data were recorded for all animals, including weight (nearest gram), head-body length from snout to vent (nearest 0.5 cm), sex, and breeding condition. During the first screening session (pre-release for reintroduced animals; first capture for subsequent offspring), all animals had a unique Trovan ID-100 tag inserted (Trovan Ltd., Douglas, UK).

During sampling 50 microlitres (µl) of blood were collected via venepuncture of the lateral tail vein using a 23G needle into a plain capillary tube for the NBT test in vitro; two smears were made for each individual. Finally, a multivette (Multivette 600 K3E, Starstedt, Germany) was attached to the needle to obtain a blood sample which was immediately refrigerated in the field, before being centrifuged in the laboratory. Fifty µl of serum were aliquoted from each sample and frozen at −20°C before corticosteroid analysis. NBT blood slides were stained following standardised protocol (see Sigma Aldrich N6876) in the lab. One hundred neutrophils were counted under an oil immersion ×100 microscope on each slide, and a mean ratio of activated to non-activated cells recorded.

Wherever possible fresh faeces were collected directly from the trap and refrigerated immediately before freezing at −20°C. On the rare occasion that no faeces were available from the trap, a warm water enema was administered and faeces would later be collected from the animals’ recovery container. All faecal and serum samples were sent on dry ice to the University of Veterinary Medicine, Vienna, for corticosteroid analysis. Faecal corticosterone was extracted using the Tetrahydrocorticosterone enzyme immunoassay [45] and plasma corticosteroid following [46].

Urine was obtained wherever possible by external manual palpation of the bladder. A reagent strip (Multistix 10 sg, Bayer Healthcare, Newbury, UK) was used to measure specific gravity, and a hand refractometer (Urine Specific Gravity Refractometer (2722), Atago, Tokyo, Japan) to give a precise measure of the hydration of each animal in refractive light units (nD).

A body condition score was developed to assess fatty reserves over the pelvic region [34], [47] (1 =  emaciated animal, individual vertebrae could be felt; 5 =  fat animal, unable to feel any vertebrae). Both hind pasterns were measured from the base of the heel to the tip of the outside toe, using digital callipers to allow investigation of fluctuating asymmetry. Although a rigorous sampling protocol was followed, only animals that were trapped were available for sampling post-release and animal welfare considerations dictated that it was not always possible to collect all samples listed per individual, due to animal size or bad weather. All animals were anaesthetised within two minutes of the trap being opened, with the entire sampling procedure being conducted in less than eight minutes.

### Statistical Analyses

We examined the relationships between each dependent stress measure (plasma corticosterone; faecal corticosterone; urine refractive index; urine specific gravity; body condition, weight/length, weight, NBT, parasite score and fluctuating asymmetry. The latter was assessed between-groups only), firstly for captive-bred voles at different time-points, and then for all adult animals to investigate differences between captive-bred and wild-born voles.

Changes in stress measures in captive-bred and reintroduced voles were tested initially using paired t-tests comparing pre-release and the first month post-release; pre-release and the last month post-release; and first and last month post-release. Repeated measures analysis of variance (ANOVA) including individual and time was not possible due to the incomplete trapping records, and for the same reason ordination could not be used to summarise the responses. The last month’s post-release data used were collected at either four months or five months, depending upon weather conditions during the fifth trapping session at a given site/year; during poor weather sampling was suspended for welfare reasons, and only those samples routinely obtainable without the use of anaesthesia were collected.

Two-way between-groups ANOVA tests were conducted to explore variation among measures of stress between captive-bred and wild-born voles, analysing each dependent variable separately. The independent variables used were session (time), sex, the origin of each animal (captive-bred or wild-born), age (adult or juvenile) and site quality, investigating the main effects and two-way interactions between them. Site quality was a score developed according to the amount of forage vegetation available at each site (see [25] for further details on calculation of forage availability). Scores ranged from 1: good forage availability (>150 cm^2^ vegetation abundance per meter of bank); 2: moderate forage availability (100–149 cm^2^ vegetation abundance) and 3: poor forage availability (<99 cm^2^ vegetation abundance). Separate models were constructed using data from capture Session 2 onwards (when both captive-bred and wild-born voles were present) for adult animals only, excluding pregnant or lactating females. Residuals were examined to ensure the data fulfilled the assumptions of the model: where appropriate dependent variables were transformed to stabilise the variance or to fulfil the assumption of normality of errors. Any model for which the Levene’s p-value still fell below 0.05 had a more stringent significance value of p = 0.01 applied to the results to account for any violation of the homogeneity of variances assumption of the test. All significant interactions between fixed factors were scrutinised before interpreting the main effects. The Tukey HSD test was used for post-hoc tests. All analyses were conducted using SPSS version 16.

### Ethics Statement

This work was part of a larger study on the reintroduction of water voles, approved by the Zoology Ethical Review Committee, a subsidiary of Oxford Universities Animal Care and Ethical Review (ACER) Committee. Work was carried out under Home Office Licence 30/2318.

## Results

In total, 422 individual animals were investigated, of which 270 were captive-bred, and 152 were wild-born (table 1). Individuals wild-born post-release were entering traps and thus available for sampling from two months post-release. From all individuals sampled, 20 were sampled on all six occasions (pre-release and each month for 5 months post-release); 16 on five different occasions; and 22 on four different occasions, all of which were from the original captive-bred cohort. Forty animals were sampled on three occasions; 76 sampled twice and 248 sampled only once, of which 156 were sampled pre-release but not recaptured. There was no significant difference in any pre-release measure between those animals which were subsequently recaptured, and those animals which were not (p≥0.059 in all cases). Animals spent a maximum of 16 hrs in the trap overnight, with handling times (from opening the trap to the animal being fully anaesthetised) being less than two minutes.

**Table 1:** Number of animals sampled pre-release and in each subsequent post-release session.

Session	Captive-bred	Wild-born	Total No animals
Pre-release (0)	270	–	270
Recapture 1	101	–	101
Recapture 2	73	49	122
Recapture 3	58	76	134
Recapture 4	45	73	118
Recapture 5	26	51	77

### Differences between pre- and post-release in captive-bred voles (see table 2 for results)

**Table 2:** Paired-t-tests for each stress measurement at different time periods; pre-release (0), first recapture (1) and last recapture (4 OR 5). Captive-bred animals only.

Stress measure (pre-release (0),first capture (1), last capturesession (4 OR 5))		Plasma Cort(0,1,4)	Faecal Cort(0,1,5)	Urine Ref Index (0,1,4)	Urine SG (0,1,4)	Fat Reserves (0,1,4)	Weight/Length (0,1,5)	Weight (0,1,5)	NBT (0,1,4)	Para Score (0,1,5)
**Pre-release - 1st recap**	**N**	26	59	21	14	42	79	94	52	79
	**t**	3.119	−1.045	4.75	0.000	3.77	−3.878	−8.516	−0.394	−1.495
	**P**	**0.005** *	0.3	≤0.001***	1.00	≤0.001***	≤0.001***	≤0.001***	0.696	0.139
	**Partial eta2**	0.28	–	0.53	–	0.26	0.16	0.44	–	–
**Pre-release - last recap**	**N**	7	26	14	4	10	15	25	37	16
	**t**	5.605	−1.034	3.692	−1.73	2.714	−0.159	−2.979	1.705	−1.464
	**P**	≤0.001***	0.311	0.003**	0.182	0.024**	0.876	0.007**	0.097	0.164
	**Partial eta2**	0.82	–	0.051	–	0.45	–	0.27	–	–
**1st recap - last recap**	**N**	15	24	6	3	27	11	22	19	12
	**t**	0.329	−1.435	−0.338	1.00	3.969	−0.103	−0.015	3.002	1.483
	**P**	0.747	0.165	0.749	0.423	≤0.001***	0.920	0.988	0.008**	0.166
	**Partial eta2**	-	–	–	–	0.38	–	–	0.033	–

* = significant at the 0.05 level; ** = significant at the 0.01 level; *** = significant at the 0.001 level.

#### Neuroendocrine

Plasma corticosterone levels decreased between pre-release (mean = 68.83, SD 72.61) and the first capture (mean = 19.75, SD = 28.97), and again between pre-release (mean = 62.88, SD = 22.68) and the final capture (mean = 9.28, SD = 6.13). There was no evidence for any differences between post-release sampling sessions.

#### Cell function

Urine refractive index decreased between pre-release (mean = 1.34, SD = 0.005) and the first recapture (mean = 1.33, SD = 0.001), and pre-release (mean = 1.34, SD = 0.003) and the final recapture (mean = 1.33, SD = 0.001) but not between recaptures, indicating that the voles become more hydrated post-release.

#### Condition indices

The fat reserve index decreased between all three sampling sessions, with the effect being greatest over the greatest time period; pre-release (mean = 3.5, SD = 0.747) to the last recapture (mean = 2.74, SD = 0.903).

Weight/length ratio increased significantly between the pre-release (mean = 10.07, SD = 2.6) and first recapture (mean = 11.26, SD = 1.76), but was not statistically significant for any other time period.

Weight increased post-release; pre-release (mean = 188.28, SD = 43.47) and first capture (mean = 223.73, SD = 37.55) and pre-release (mean = 191.96, SD = 45.75) and final capture (mean = 228.4, SD = 34.21). There was no evidence for variation between post-release sessions.

#### Immune system function

NBT showed a statistically significant decrease only between the first (mean = 75.08, SD = 8.40) and final recapture (mean = 65.12, SD = 12.41).

### Differences between captive-bred and wild-born groups (see table 3 for results)

**Table 3:** Two-way between groups ANOVA.

	Log Plasma Cort			Log FaecalCort			Log Urine Ref Index			Urine SG			Fat Reserve Index		
	F _1,142_	P	Partial eta^2^	F _1,189_	p	Partial eta^2^	F _1,91_	p	Partial eta^2^	F _1,43_	p	Partial eta^2^	F _1,208_	p	Partial eta^2^
**Session** * **Origin**	0.92	0.4		1.33	0.267		1.19	0.319		0.77	0.472		0.45	0.719	
**Sex** * **Origin**	0.02	0.88		3.12	0.079		4.54	**0.036** *	0.057	1.19	0.283		1.02	0.315	
**SQ** * **Origin**	1.3	0.276		0.23	0.796		0.159	0.211		1.3	0.264		1.33	0.268	
**Session** * **Sex**	3.16	**0.046** *	0.047	0.67	0.571		2.86	**0.043** *	0.103	1.01	0.376		0.43	0.735	
**SQ** * **Session**	2.09	0.085		0.47	0.801		1.87	0.143		12.38	≤0.001***	0.421	0.24	0.945	
**SQ** * **Sex**	1.2	0.304		1.02	0.363		4.01	**0.022** *	0.097	2.47	0.125		0.79	0.454	
**SQ**	1.4	0.25		5.32	0.006**	0.058	0.85	0.432		0.5	0.486		0.38	0.687	
**Session**	1.69	0.189		4.61	0.004**	0.074	1.35	0.264		8.19	0.001***	0.325	1.88	0.135	
**Sex**	3.34	0.07		0.06	0.807		0.34	0.564		3.59	0.067		3.61	0.059	
**Origin**	3.86	0.052		0.05	0.818		2.23	0.14		1.25	0.271		31.3	≤0.001***	0.14
	**Weight/Length**			**Weight**			**Log NBT**			**Ectoparasite score** $			**Log Fluctuating Asymmetry**		
	**F _1,209_**	**P**	**Partial eta^2^**	**F _1,261_**	**p**	**Partial eta^2^**	**F _1,177_**	**p**	**Partial eta^2^**	**F _1,209_**	**p**	**Partial eta^2^**	**F _1,201_**	**p**	**Partial eta^2^**
**Session** * **Origin**	0.5	0.682		2	0.115		0.09	0.914		0.4	0.752		1.15	0.332	
**Sex** * **Origin**	0.05	0.823		0.08	0.771		9.42	0.003**	0.055	8.27	0.005**	0.042	0.86	0.357	
**SQ** * **Origin**	0.31	0.736		0.46	0.633		1.36	0.259		0.33	0.716		1.06	0.349	
**Session** * **Sex**	1.78	0.153		2.01	0.113		2.5	0.089		0.16	0.926		0.44	0.728	
**SQ** * **Session**	0.39	0.855		0.22	0.956		1.44	0.223		0.47	0.795		0.74	0.595	
**SQ** * **Sex**	0.19	0.828		1.48	0.231		0.8	0.452		0.15	0.863		1.27	0.284	
**SQ**	1.17	0.311		4.06	0.018**	0.033	1.32	0.271		0.45	0.642		1.33	0.266	
**Session**	0.32	0.812		0.71	0.549		0.02	0.979		1.17	0.328		0.38	0.771	
**Sex**	2.34	0.128		3.61	0.059		0.01	0.948		6.67	0.011		0.44	0.51	
**Origin**	51.8	≤0.001***	0.214	104.28	**≤0.001**	0.302	6.5	**0.012**	0.039	3.44	0.065		0.6	0.438	

$ More stringent value of *p* = 0.01 applied to account for lack of homogeneity of variances assumed by the test, indicated by a Levene’s value <0.05.

* =  significant at the 0.05 level; ** =  significant at the 0.01 level; *** =  significant at the 0.001 level.

All Adult animals from capture session two onwards (when both captive-bred and wild-born are present; excluding pregnant or lactating females), sex, origin, site quality and session. Only two-way interactions and main effects are considered.

Four stress measures were influenced by origin: fat reserves (F_1,211_ = 43.21, p≤0.001), with captive-bred voles having lower fat reserves (estimated marginal mean = 2.93, SE = 0.071) than wild-born voles (mean = 3.71, SE = 0.96); weight/length ratio (F_1,212_ = 180.96, p≤0.001) with captive-bred voles having a greater weight/length ratio (estimated marginal mean = 11.13, SE = 0.191) than wild-born voles (mean = 7.93, SE = 0.191); weight (F_1,263_ = 256.94, p≤0.001) with captive-bred voles being heavier (estimated marginal mean = 226.16, SE = 3.125) than wild-born voles (mean = 147.69, SE = 3.77) and NBT (F_1,180_ = 10.88, p = 0.001) with captive-bred voles having higher NBT measures (estimated marginal mean = 69.09, SE = 1.023) than wild-born voles (mean = 63.49, SE = 1.36).

#### Neuroendocrine

There was evidence for an interaction between session and sex in predicting plasma corticosterone levels. Inspection of the means suggested a sex difference only in Session 2 where females had a higher level than males (mean = 4.93, SE = 0.31, compared with mean = 4.15, SE = 0.15).

No interaction effects were found for predicting faecal corticosterone levels. Session and site quality were both statistically significant predictors. Post-hoc comparisons using the Tukey HSD test indicated that faecal corticosterone levels were significantly lower in site quality one (estimated marginal mean = 2.58, SE = 0.12) compared to both site qualities two (mean = 3.11, SE = 0.13) and three (mean = 3.29, SE = 0.25), which showed no difference between them. Faecal corticosterone levels in Session 5 were also significantly lower (estimated marginal mean = 3.2, SE = 0.21) than for all other sessions.

#### Cell function

The effect of session, site quality and origin all varied between sexes (interaction effects). Splitting the data by sex and re-running the analyses found site quality to have a statistically significant impact on male hydration levels (F_2,40_ = 7.28, p = 0.002) with post-hoc tests showing that male hydration levels were significantly lower between site qualities one and three, and two and three.

The interaction between site quality*session was significant for urine specific gravity thus the data were split by session and analyses repeated. Session 2 showed a significant effect for site quality (F_1,15_ = 44.35, p≤0.001) with post-hoc analyses indicating that animals were significantly more hydrated in site quality one (mean = 1.007, SE = 0.002) than site quality three (mean = 1.015, SE = 0.002) (no data were available for site quality two). Sessions 3 and 4 showed no significant effects for site quality.

#### Condition indices

Animal origin had a significant effect on fat reserves of water voles; wild-born animals had more pelvic fat stores (estimated marginal mean = 3.85, SE = 0.134) than did captive-bred individuals (mean = 2.85, SE = 0.104). A similar pattern was found for weight/length ratio; captive-bred animals had a greater weight/length ratio (estimated marginal mean = 11.07, SE = 0.212) than did wild-born (estimated marginal mean = 8.17, SE = 0.272).

Both origin and site quality were significant predictors of weight. Captive-bred animals were heavier (mean = 228.9, SE = 4.55) than wild-born (mean = 152.0, SE = 5.28), with post-hoc analyses indicating that animals in site quality two were significantly heavier than in either site qualities one or three (site quality one mean = 182.6, SE = 3.31; site quality two mean = 198.9, SE = 5.16; site quality three mean = 189.6, SE = 9.45).

#### Immune system function

There was a significant interaction of sex*origin for NBT levels. Closer inspection by splitting the data by sex showed that captive-bred males had significantly higher NBT levels (estimated marginal mean = 4.28, SE = 0.027) than wild-born males (estimated marginal mean = 4.019, SE = 0.058). There was no effect for females.

Similarly, there was a significant interaction of sex*origin for parasite score: splitting the data by origin revealed that captive-bred males had higher parasite scores (estimated marginal mean = 1.9, SE = 1.33) than wild-born males (estimated marginal mean = 1.07, SE = 1.72).

## Discussion

We demonstrate that different measures of stress can be affected by different environmental and physiological factors, and an assessment accounting for all of them is desirable for both welfare and for monitoring the efficiency of reintroductions. Combining different measures of stress provides a more complete indication of the stress response of an individual at any time and of the diverse biological systems perturbed by different stressors, and we therefore recommend that measures from different physiological systems are utilised simultaneously wherever possible. This can indicate areas of the reintroduction process that require refinement to help reduce stress and improve animal welfare.

### Pre- and Post-release Differences

Captive-bred reintroduced animals clearly varied in their levels of stress between pre-release and both short- and long-term post-release sessions. Of the different methodologies used only faecal corticosterone, urine specific gravity and parasite score showed no statistically significant change over time.

Captive-bred animals tended to have lower stress responses than wild-born voles (they were heavier, had greater weight/length ratios and higher NBT scores, despite having lower levels of body fat). These findings suggest that the captive-bred voles were either more muscular or had greater bone density, possibly attributable to a better quality of nutrition whilst they were maintained in captivity [48]. The effects of sex on stress varied both with origin and with site quality. Captive-bred males had greater NBT levels suggesting greater immunocompetence, and all male water voles were less hydrated in poorer quality habitat. These observations possibly support the testosterone immunoredistribution hypothesis which predicts a trade-off between stress and suppression of the entire immune system by redistributing immune cells to sites of potential injury during mating rituals [49]. The larger ectoparasite burden of the captive-bred males does not necessarily indicate a reduced level of immunocompetence: while such a negative relationship has been demonstrated in bird species [40], studies on small mammals have shown that although males have higher ectoparasite infection levels than females, there is no evidence of male biased mortality as a result [50].

### Neuroendocrine Differences

We observed a reduction in plasma corticosterone levels post-release, indicating a decrease in chronic stress levels over time; a result in accord with faecal glucocorticoid measurements from whooping cranes undergoing reintroduction [51]. Fletcher and Boonstra [52] found that the time spent in a live capture trap did not affect corticosterone levels in meadow voles *Microtus pennsylvanicus a*nd so it is appropriate to treat trapping as a constant within this study. The anaesthesia process in our study was short enough (under two minutes) to preclude corticosterone being released because corticosterone release takes at least three minutes [53], [54]. Therefore changes in plasma corticosterone levels were unlikely to be altered by the handling process [55].

Animals at higher quality sites had lower faecal corticosteroid levels. Site quality is known to have a significant impact on food availability and growth rates of water voles [43]. Ensuring site quality is of the highest possible quality before release is likely to minimise stress in reintroductions generally. Because circulating corticosteroids are integrated into faeces over a period of time they are less affected by episodic fluctuations of hormone secretion and might therefore present a better reflection of the hormonal status of an individual [56] than plasma corticosteroid levels.

### Changes in Cell Function

Water voles became more hydrated post-release, as indicated by urine refractive index. Their captive cages (between the outdoor breeding enclosures and reintroduction) could not accommodate water bowls. Some vole-breeders claim apple supplies sufficient water and do not supply water bottles (pers. obs.); these were provided for the period during which they were housed at Oxford. It is therefore unsurprising that hydration increased post release. However, whether the animals were clinically dehydrated, or simply less-well hydrated, hydration status remains an important factor for cell function [33].

### Condition Indices

As expected, all three condition indices showed significant changes post-release: captive-bred voles increased in size but decreased in body condition over time as simultaneously these animals had their first opportunity to breed, potentially resulting in lower fat reserve storage. This trend continued, presumably because water voles produce many litters throughout the season. This was less clear for wild-born voles, possibly due to a paucity of long-term data for adult animals. We are currently unable to tease out whether captive-bred body condition is significantly influenced by animal origin, age, or by the actual reintroduction process. An alternative hypothesis is that the captive-bred voles may have carried excess fat in captivity due to an absence of predators, but upon release may become slimmer to aid escape potential, a mechanism which has previously been shown in great tits *Parus major* with perceived increased predation risk by a model sparrowhawk [57].

### Immune System Function

Immunocompetence increased over time, reflecting environmental acclimatisation [17]. There was a much stronger effect for captive-bred than wild-born males, suggesting that those captive-bred voles that survived longer post-release were more able to cope with the novel environment, despite their decrease in fat reserves post-reintroduction.

Captive-bred males had significantly higher parasite scores throughout, which may reflect the lack of competition between captive-bred and wild-born animals due to their disparate sizes. It is however plausible that as the wild-born males increased in size and associated movement over time, so their parasite-encounter rate and burden will also increase, but this does not necessarily have a negative impact on the individuals concerned.

### Conclusions and Implications for Future Reintroductions

There are few published studies which investigate the impact of stress throughout a reintroduction program (e.g. [7], [17], [51], [58]), and fewer still which use a combination of different methodologies. We investigated four categories of welfare measures in this study; neuroendocrine, cell function, body condition and immune system function, many of which produced varying results throughout the reintroduction process, demonstrating that different biological responses were elicited by different independent variables. Our study highlights the need for the use of multiple measures, and that relying upon a single measure of stress as an indicator of animal welfare, or even measures within just one category, are unlikely to reveal the full impact that the reintroduction process has upon the various biological functions required to maintain a healthy individual. Nonetheless, we demonstrate that there are a number of tools available which may be used simultaneously for monitoring the impact of different methodologies on individuals, allowing the reintroduction biologist to refine their protocols accordingly.

These findings have implications for future reintroductions, highlighting causative factors of stress that might be avoided in future studies (e.g. water availability and pre-release hydration status). The specific drivers and causes of stress within any individual reintroduction programme will depend upon a number of factors, not least the species of concern, but also the housing, transportation and release methods adopted. This study demonstrates a number of methods which offer a feasible way of monitoring stress during reintroduction, specifically for small mammals but also applicable to other species, are available and highlights the importance of considering a combination of methods when determining animal welfare for any species undergoing a reintroduction.
